# Novel Low-Crystallinity Polyetheretherketone Copolymers for 3D Printing

**DOI:** 10.3390/polym18050558

**Published:** 2026-02-25

**Authors:** Azamat Zhansitov, Zhanna Kurdanova, Kamila Shakhmurzova, Azamat Slonov, Azamat Khashirov, Elena Rzhevskaya, Khasan Musov, Alanbek Tlupov, Svetlana Khashirova

**Affiliations:** Center for Progressive Materials and Additive Technologies, Kabardino-Balkarian State University Named After H.M. Berbekov, 360004 Nalchik, Russia; kurdanova09@mail.ru (Z.K.); shahmurzova.kamila@yandex.ru (K.S.); azamatslonov@yandex.ru (A.S.); khashaz@yandex.ru (A.K.); elena.r-1382@mail.ru (E.R.); xmusov@gmail.com (K.M.); tlupovaslanbek99@gmail.com (A.T.); new_kompozit@mail.ru (S.K.)

**Keywords:** polyetheretherketone, 4,4′-dichlorodiphenylsulfone, copolymers, polycondensation, mechanical properties, crystallization, 3D printing

## Abstract

To improve the quality of additive manufacturing of PEEK parts, copolymers with varying 4,4′-dichlorodiphenylsulfone (DCDPS) contents were synthesized. A study of the thermophysical properties of the resulting copolymers revealed that increasing the DCDPS content leads to lower melting temperatures, crystallization temperatures, and degree of crystallinity, while simultaneously increasing the glass transition temperature. It was found that structural amorphization leads to a predictable decrease in the strength and elastic modulus of both cast and printed samples. However, at a DCDPS concentration of 15%, the decrease in mechanical properties is offset by an increase in polymer chain rigidity. The practical result of this study was the successful adaptation of the material to FDM printing: copolymers with DCDPS contents in the range of 5–20% ensured stable molding without deformation or delamination, demonstrating an optimal balance between processability and performance.

## 1. Introduction

Three-dimensional (3D) printing, as a dynamically developing additive manufacturing technology, opens broad prospects for modern industry. Its key advantages include a significant reduction in production costs, a high degree of design freedom, and the possibility of rapid prototyping of products with complex geometric configurations [1,2,3]. Due to these characteristics, 3D printing technology has become widespread in such strategically important industries as the aerospace industry, automotive manufacturing, architectural design, and biomedical engineering [4,5,6,7]. Currently, among the many existing methods of three-dimensional printing, a special place is occupied by Fused Deposition Modeling (FDM) technology. This method has proven itself as one of the most sought-after and cost-effective approaches, especially when working with thermoplastic polymers of predominantly amorphous structure: polylactic acid (PLA) [8,9], acrylonitrile butadiene styrene (ABS) [10,11], polycarbonate (PC) [12,13], and polyamide (PA) [14,15]. Nevertheless, despite the processability of the printing process, traditional amorphous thermoplastics possess limited performance properties and do not fully meet the stringent requirements of specialized engineering applications. In this regard, polyetheretherketone (PEEK)—a high-performance engineering polymer with outstanding performance characteristics—is of particular interest. PEEK is capable of maintaining stable mechanical properties under significant loads and demonstrates exceptional thermal resistance under elevated temperature conditions. Due to its unique complex of performance characteristics, the 3D printing technology of polyetheretherketone products has attracted significant attention from researchers and engineers working in high-tech application areas. Particular interest in PEEK is shown by specialists in aerospace, nuclear, and biomedical engineering, where increased requirements for thermomechanical stability and the reliability of structural materials are imposed [16,17,18,19].

The practical implementation of FDM printing of PEEK products is associated with significant technological difficulties due to the specific physicochemical properties of this polymer. A key problem is the need to process the material at extremely high temperatures in the range of 350–440 °C (with a minimum threshold > 370 °C) [20,21,22,23]. An additional complexity is the high degree of crystallinity of the polymer and its tendency toward uncontrolled crystallization during the fused deposition process [21]. Rapid and uneven cooling of the material causes significant internal thermal stresses, which manifest as warping and geometric deformations of finished parts, especially in the manufacture of large-scale or thin-walled structures. The combination of these factors leads to the fact that products manufactured by the FDM method from PEEK without proper process optimization are characterized by a number of structural defects: increased porosity, insufficient interlayer bonding strength, and the presence of microcracks within the material volume [21]. These defects significantly reduce the mechanical properties of the final products compared to injection-molded specimens.

An effective solution to this problem is the use of modified PEEK grades with slow crystallization kinetics. Reducing the rate of ordering of the supramolecular structure allows for an expansion of the temperature-time window during which polymer chains retain the mobility necessary for interdiffusion and the formation of entanglements at the interlayer boundary. This is achieved primarily through copolycondensation: the introduction of specific comonomers disrupts the conformational regularity of the polymer, preventing dense chain packing and lowering the melting and crystallization temperatures.

Analysis of modern research reveals several effective strategies for such modification. In particular, Victrex is actively developing a method involving the introduction of rigid biphenyl fragments into the chain (formation of PEEK/PEDEK copolymers). It has been experimentally confirmed that the presence of polyetherbiphenyletherketone units allows for a reduction in T_m_ to values around 300 °C and a significant slowing of crystallization kinetics [24]. This ensures improved layer adhesion, resulting in samples printed in a vertical orientation demonstrating significantly higher strength characteristics compared to PEEK homopolymer, since the solidification process does not block diffusion in the early stages of cooling.

An alternative approach is the targeted disruption of macromolecule symmetry through the use of meta-substituted monomers. Replacing part of the para-oriented 4,4′-difluorobenzophenone with meta-oriented 3,5-difluorobenzophenone creates angular kinks in the chain, which hinders crystallization [25]. At a comonomer ratio of 80:20, the melting temperature decreases from 316 °C to 284 °C, which not only facilitates processing but also promotes the formation of a more homogeneous material structure.

The most significant improvement in the mechanical properties of 3D-printed parts was achieved by introducing bulky side groups, such as fluorene (carded) fragments, into the PEEK structure [26]. Modifying the polymer with 10 mol% fluorene groups increases the interlaminar strength to 41 MPa, which is comparable to specialized commercial grades. A further increase in the modifier content to 15 mol% leads to an increase in tensile strength to 67 MPa and relative elongation to 11.23%. These values exceed those of pure PEEK by 400–500%, which is explained by the formation of a structure with a high proportion of amorphous phase at the layer boundaries, ensuring deep interdiffusion of macromolecules. Thus, kinetic control of the crystallization process through chemical modification of the chain is a determining factor in ensuring the integrity and strength of polyarylene ether ketone products produced by 3D printing.

Implementing this approach through copolymer synthesis allows for a controlled reduction in the proportion of the crystalline phase, thereby adapting the material to the conditions of layer-by-layer fused deposition. Unlike most existing studies, which focus primarily on the creation of composite materials or optimization of printing conditions for commercial grades, the scientific novelty of this work lies in the targeted chemical modification of the PEEK backbone by introducing bulky sulfone fragments. This modification strategy, compared to known analogs, offers a new way to control crystallization kinetics without critical loss of thermal stability, which remains a poorly understood aspect of materials science for additive manufacturing. Therefore, the goal of the study is to synthesize a sulfo-containing polyetheretherketone with an optimized amorphous phase to ensure stable and uniform 3D printing.

## 2. Materials and Methods

### 2.1. Materials

The following were used in the work: diphenyl sulfone (DPS) of (99.9%) was obtained from Shandong Zhishang Chemical Co., Ltd. (Jinan, China), 4,4′-difluorobenzophenone of (99.94%) was obtained from Shandong Zhishang Chemical Co., Ltd. (Jinan, China), hydroquinone (99.99%) was obtained from Shandong Zhishang Chemical Co., Ltd. (Jinan, China), 4,4′-dichlorodiphenyl sulfone (≥99.5%) was purchased from Hebei Jianxin Chemical Co., Ltd. (Cangzhou, China), Potassium carbonate (99+%) was purchased from Reachem (Moscow, Russia), Sodium carbonate (99+%) was purchased from Reachem (Moscow, Russia), and acetone (99+%) was purchased from EKOS-1 (Moscow, Russia).

### 2.2. Synthesis of PEEK and Copolymers

**Scheme 1:**
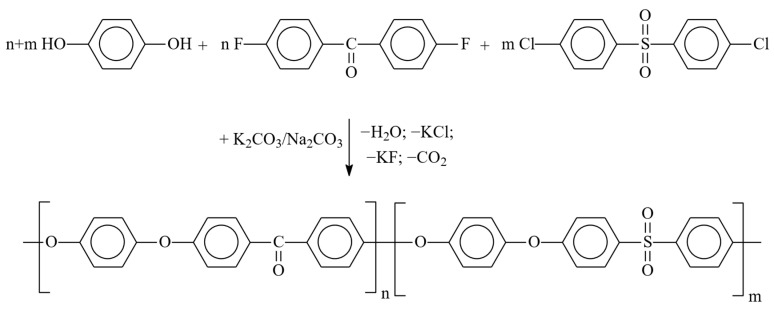
Synthesis of PEEK copolymer.

The synthesis of polyetheretherketone and copolymers was carried out by the polycondensation method via the nucleophilic substitution reaction in accordance with the procedure described in [7] at different ratios of the monomers 4,4′-difluorobenzophenone, 4,4′-dichlorodiphenylsulfone and hydroquinone (Table 1) in the presence of sodium carbonate and potassium carbonate in a ratio of 0.2:0.8 in diphenylsulfone (Scheme 1) in a stream of inert gas with continuous stirring while gradually increasing the temperature to 305–320 °C. After 1–6 h of synthesis at 320 °C, the reaction mixture was unloaded, cooled and the resulting solid was ground. Diphenylsulfone and inorganic salts were removed by washing successively with acetone (twice), water (three times) and acetone (once). The resulting polymer powder was dried at 120 °C in vacuum for 12 h.

### 2.3. Characterization and Methods

IR spectra were recorded on a Fourier spectrometer (Spectrum Two; PerkinElmer, Inc., Waltham, MA, USA) in the range of 4000–450 cm^−1^ with a spectral resolution of 0.4 cm^−1^.

The melt flow index (MFI) was determined on a PTR Lab-02 capillary viscometer (LOIP, Saint Petersburg, Russia) at 380 °C and a load of 5 kg.

Mechanical testing was performed on a Gotech Testing Machine CT-TCS 2000 (Taichung Industry Park, Taichung City, Taiwan) at 23 °C. The average value obtained from testing 5 samples was taken as the result of the analysis.

Differential scanning calorimetry analysis was performed according to GOST R 55134-2012 on a DSC 4000 instrument (PerkinElmer, Inc., Waltham, MA, USA) in an inert atmosphere over a temperature range of 30–370 °C. The scanning rate was 10 °C/min. The glass transition and melting temperatures obtained during the second heating of the specimen were used as the analysis results. Prior to testing, the polymer specimens were dried in a vacuum oven in several stages at various temperatures, ranging from 90 to 150 °C.

The study of the dependence of the rheological properties of composites on frequency was carried out on a rotational rheometer MCR 302 (Anton Paar, Graz, Austria), at γ = 1% and in the range of angular velocity from 1 to 620 rad/s at 380 °C.

### 2.4. FDM Printing

3D printing filaments were produced on a laboratory Twin Tech Screw 10 mm twin-screw extruder (UK).

3D printing of the synthesized PEEK and copolymers was performed on a VOLGOBOT A4 Pro 3D printer (Russia) using the following print modes: layer height 0.2 mm, layer width 0.4 mm, 45/45 linear stackup, infill density 100%, flow factor 0.96, print speed 15 mm/s, nozzle temperature 460 °C, bed temperature 235 °C, and chamber temperature 235 °C.

## 3. Results and Discussion

### 3.1. Study of the Structure of Copolymers by IR Spectroscopy

**Figure 1:**
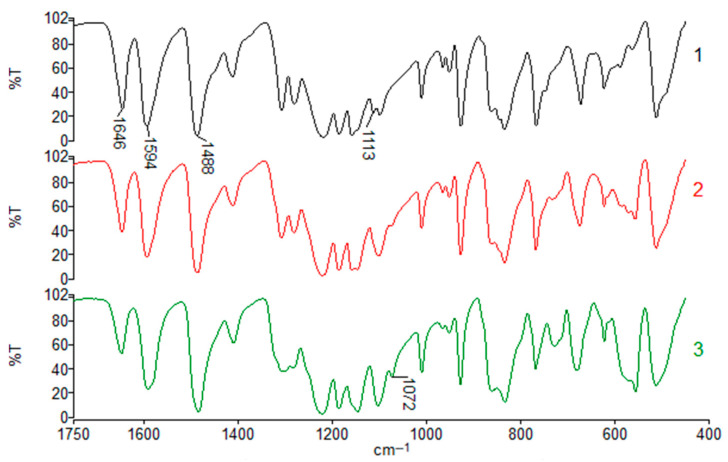
IR spectra of PEEK homopolymer (1) and its copolymers with 20% DCDPS (2), and 50% DCDPS (3).

The structure of the synthesized copolymers was studied using IR spectroscopy (Figure 1). The IR spectra of SPEEK demonstrate the presence of all the characteristic bands corresponding to this polymer. Skeletal vibrations of aromatic carbon-carbon bonds are manifested by bands with maxima at 1594 and 1488 cm^−1^. The intense absorption band at 1646 cm^−1^ corresponds to the stretching vibrations of the carbonyl group—C=O, the intensity of which decreases consistently with increasing concentration of the 4,4′-dichlorodiphenyl sulfone (DCDPS) comonomer in the SPEEK structure. The characteristic absorption peak at 1072 cm^−1^ is due to the symmetric and asymmetric stretching vibrations of the sulfone group—SO_2_, confirming the successful incorporation of DCDPS into the copolymer structure. In the IR spectrum of PEEK, the peak (or absorption band) at 1113 cm^−1^ is attributed to the stretching vibrations of the ether linkage (C–O–C). The coalescence of peaks and the significant increase in absorption intensity in the 1113 cm^−1^ region upon the incorporation of 4,4′-dichlorodiphenyl sulfone are due to the superposition of spectral lines: the band corresponding to the stretching vibrations of the ether linkage overlaps with the adjacent and intense band of the symmetric stretching vibrations of the sulfone group (-SO_2_-). The high polarity of the S=O bonds results in a significant transition dipole moment, leading to a dominant contribution of the sulfone groups to the signal amplitude and the formation of a broadened, high-intensity peak.

### 3.2. Thermal Properties

**Figure 2:**
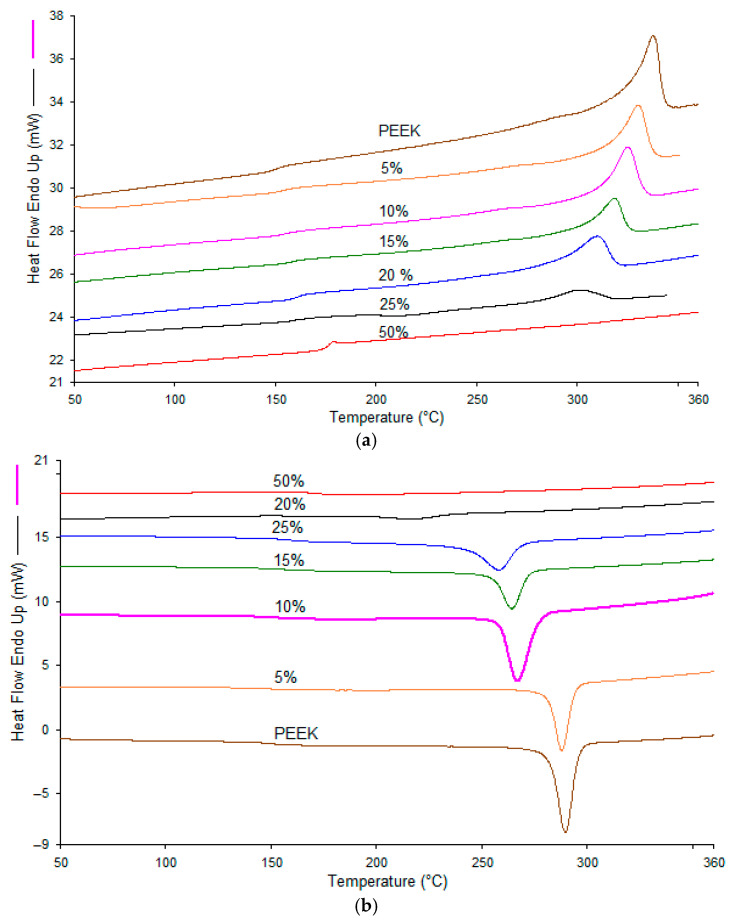
DSC thermograms: melting curves (**a**) and crystallization curves (**b**) of PEEK copolymers.

Figure 2 and Table 2 present the DSC analysis results. With increasing DCDPS content, a monotonic increase in the glass transition temperature (Tg) is observed, from 149.9 °C for the homopolymer to 176.9 °C for the copolymer with 50% DCDPS. This effect is explained by the introduction of rigid polar sulfone groups (-SO_2_-) into the polymer chain. Unlike flexible ether bonds, these groups create steric hindrance and limit the segmental mobility of macromolecules, thereby increasing the overall chain rigidity.

As Tg increases, the melting point (T_m_) and crystallization temperature, as well as the degree of crystallinity, decrease. The mechanism of this amorphization is the disruption of the regularity of the PEEK backbone by the incorporation of the comonomer. Structural heterogeneity prevents the chains from densely packing into the crystal lattice, as evidenced by the broadening of the crystallization temperature range, indicating a slowing of the kinetics of this process. At a DCDPS content of 50%, the material becomes completely amorphous, as evidenced by the absence of melting and crystallization peaks. The slight deviation from the general trend for the sample with 10% DCDPS (increased crystallinity) is due to its lower molecular weight, which facilitates chain packing.

### 3.3. Mechanical Properties

**Figure 3:**
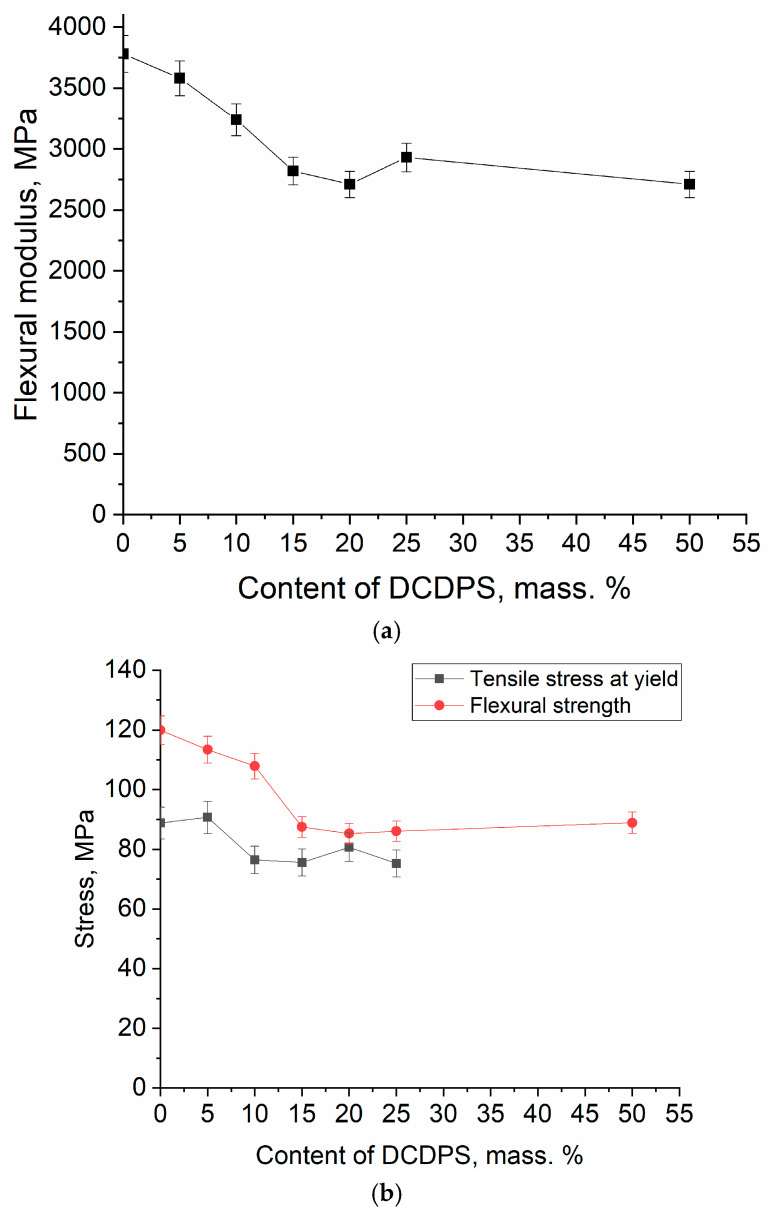
Dependence of the modulus of elasticity (**a**) and strength (**b**) on the content of DCDPS for samples obtained by injection molding.

A study of the mechanical properties of PEEK copolymers showed (Figure 3) that the addition of up to 15% DCDPS is accompanied by a uniform decrease in the flexural modulus, as well as flexural and tensile strength. A plateau is reached starting at 15%, with mechanical properties changing insignificantly up to 50% DCDPS. This initial decrease is likely due to a decrease in the material’s crystallinity. A further increase in the comonomer proportion has virtually no effect on the mechanical properties, since, despite amorphization, the addition of DCDPS increases chain rigidity, as evidenced by an increase in the glass transition temperature.

### 3.4. Rheological Properties

**Figure 4:**
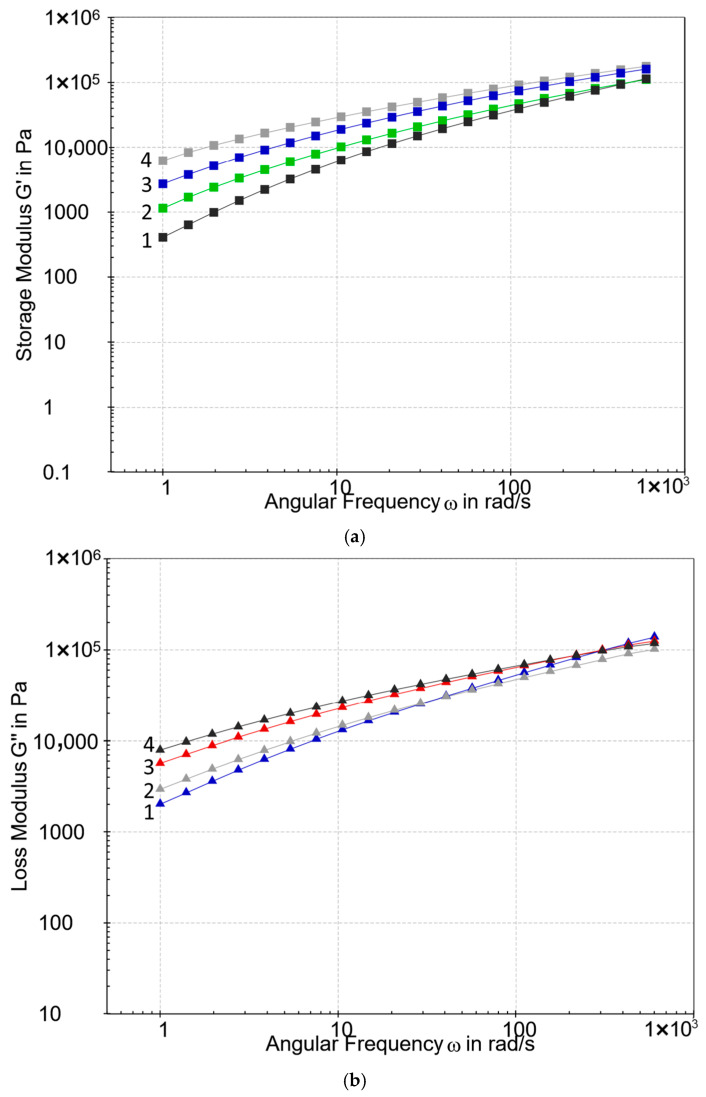
Frequency dependences of G′ (**a**) and G″ (**b**): 1—PEEK; 2—SPEEK-5; 3—SPEEK-15; 4—SPEEK-20.

The rheological properties of copolymers for 3D printing were studied. To assess the rheological state of the melt, the frequency dependences of the storage modulus (G′) and loss modulus (G″) of the PEEK homopolymer and copolymers containing 5, 15, and 20% DCDPS were measured (Figure 4). It is evident that G′ and G″ for the studied materials increase with increasing frequency, which is typical for most polymers. At low frequencies, the deformation time exceeds the relaxation time of the polymer chains, as a result of which the chains have time to relax, and the material exhibits a predominantly viscous response. As the frequency increases, the exposure time becomes shorter than the relaxation time, as a result of which the chains do not have time to rearrange, leading to an increase in the elastic component. In the low-frequency region, the differences between the modules are clearly expressed and are manifested in their consistent increase with the growth of the DCDPS content, whereas at high frequencies the values of G′ and G″ become significantly closer.

**Figure 5:**
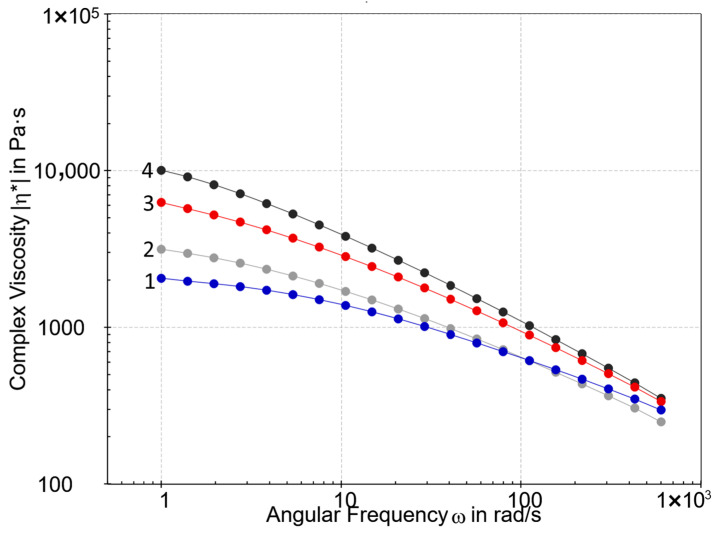
Dependence of complex viscosity on frequency: 1—PEEK; 2—SPEEK-5; 3—SPEEK-15; 4—SPEEK-20.

A study of the dependence of complex viscosity on frequency showed (Figure 5) that the melts of all materials are pseudoplastic fluids, the viscosity of which decreases with increasing angular velocity. With increasing DCDPS content, viscosity increases, which is more noticeable in the low-frequency region, while at high frequencies the differences in viscosity values are not as significant.

It is known that for most FDM printing processes, the shear rate in the nozzle is in the range of 100–250 s^−1^ [27]. To establish the relationship between the complex viscosities obtained from frequency tests and the viscosity under steady-state shear, the Cox-Merz rules (Equation (1)) can be used.

(1) $$\eta \left(\dot{\gamma \;}\right)=\eta^{*}\left(\omega \right)\;for\;\dot{\gamma \;}=\omega$$

where *η* is the viscosity under steady-state shear, η* is the complex viscosity, $\dot{\gamma \;}$ is the shear rate, and *ω* is the frequency [28]. Thus, the studied materials demonstrate fairly close viscosity values in the range from 100 to 300 rad/s, and therefore no fundamental differences in their rheological behavior during 3D printing are expected.

### 3.5. Properties of 3D Printed Samples

**Figure 6:**
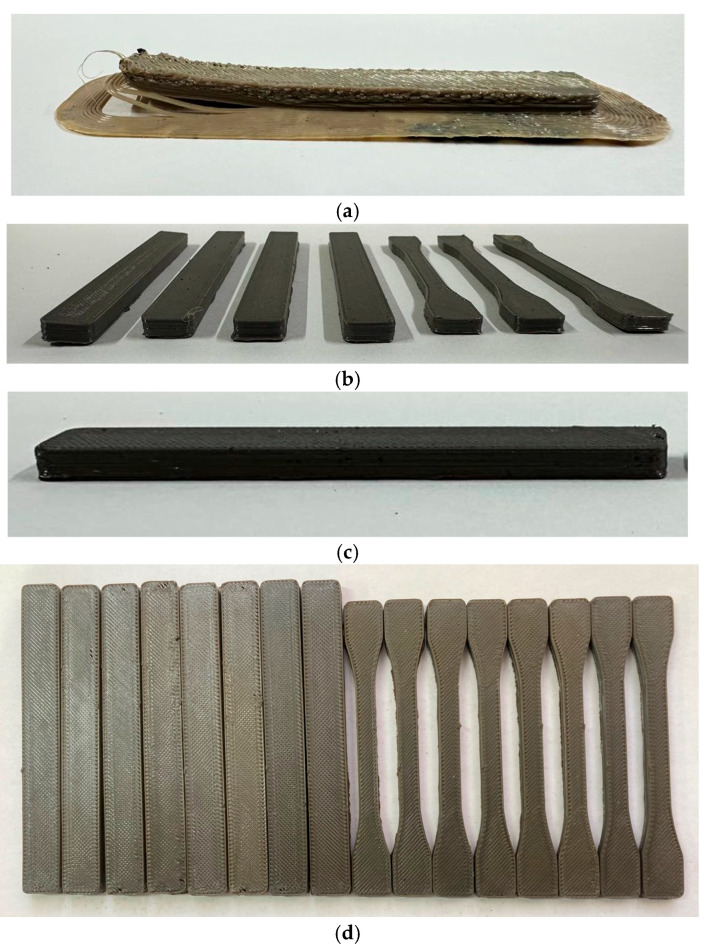
Photo of printed samples: (**a**)—PEEK; (**b**)—SPEEK-5; (**c**)—SPEEK-15; (**d**)—SPEEK-20.

Filaments for 3D printing using FDM were produced from PEEK homopolymer and synthesized copolymers containing 5, 15 and 20% DCDPS. The images (Figure 6) show that printing with PEEK homopolymer is accompanied by sample lift-off from the build plate due to crystallization processes. The raised edges of the block cause collisions between the nozzle and the printed layer, leading to defects. Adding 5% DCDPS significantly reduces shrinkage and prevents sample warping. Similarly, the copolymers containing 15 and 20% DCDPS demonstrate good print quality.

**Figure 7:**
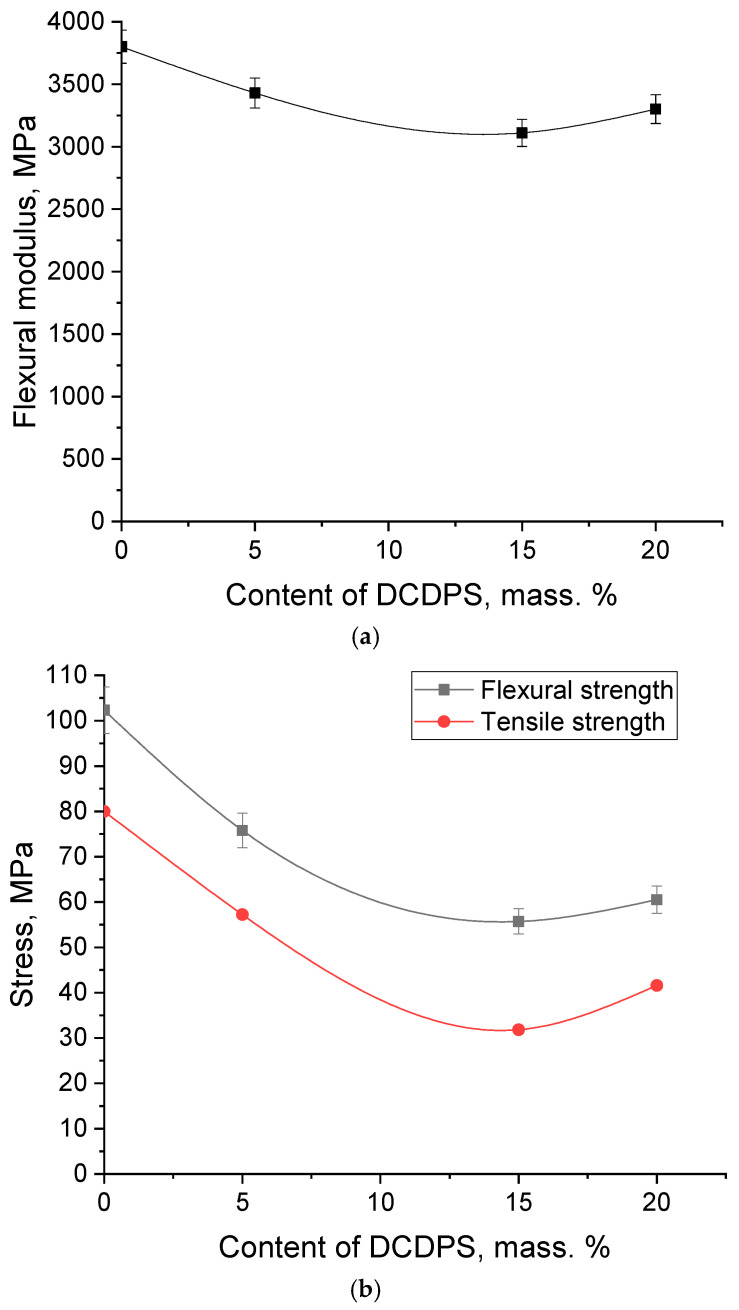
Dependence of the modulus of elasticity (**a**) and strength (**b**) on the content of DCDPS for 3D printed samples.

Figure 7 shows that for the printed samples, similar to the injection-molded samples, a decrease in the elastic modulus and strength in both bending and tensile strength is observed with increasing DCDPS content. The elastic modulus values of the printed samples are comparable to those of the injection-molded samples. The strength of the homopolymer is also close to that of the injection-molded samples, while the flexural and tensile strength of the copolymers is significantly lower. It is noteworthy that the copolymer with a 20% DCDPS content exhibits slightly higher mechanical properties compared to SPEEK-15.

**Figure 8:**
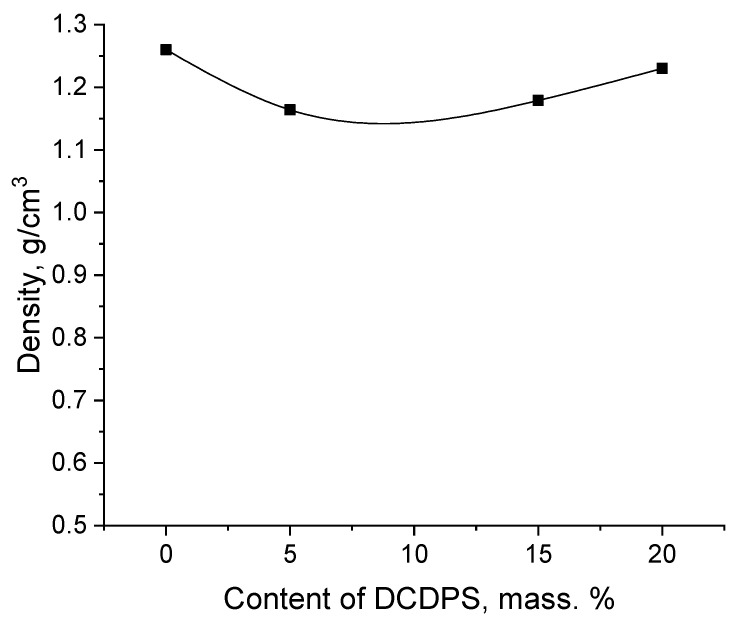
Dependence of the density on the content of DCDPS for 3D printed samples.

Figure 8 shows that the density of the printed samples follows a similar trend to their mechanical properties. The exception is the copolymer containing 5% DCDPS, which exhibits the lowest density despite superior mechanical properties compared to the other copolymers.

**Figure 9:**
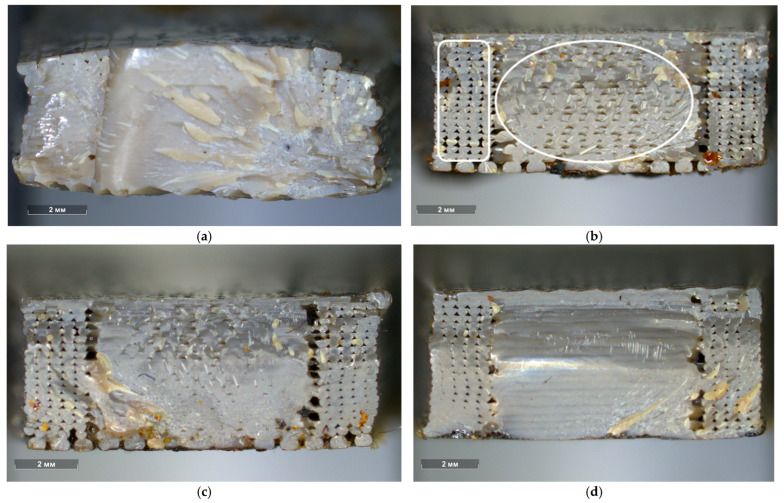
Micrographs of chips of printed samples: (**a**)—PEEK; (**b**)—SPEEK-5; (**c**)—SPEEK-15; (**d**)—SPEEK-20.

Micrographs of cleavage sections of the printed samples confirm the obtained density data (Figure 9). The printed PEEK homopolymer sample is characterized by a dense and uniform structure. In the SPEEK-5 sample, a significant number of voids are observed both in the contour region, where the threads are oriented at an angle of 0° in the longitudinal direction (Figure 9b, highlighted by a rectangle), and in the central part with a thread orientation of +45/−45° (Figure 9b, highlighted by an oval). The printed copolymer with a 15% DCDPS content is distinguished by a significantly smaller number of voids in the sample core and smaller defect sizes in the contour region. SPEEK-20 is characterized by a complete absence of voids in the central part, which determines the highest density among the copolymers and good elastic strength properties. The high mechanical properties of SPEEK-5, despite its reduced density, are apparently related to the intrinsic properties of the material. It has previously been shown that injection-molded samples of this copolymer exhibit the highest elastic-strength properties compared to other copolymers (Figure 3). Thus, the insufficient density of the printed SPEEK-5 samples is compensated for by its original characteristics, resulting in superior mechanical properties even at a reduced density.

## 4. Conclusions

Thus, it was established that the introduction of DCDPS leads to a decrease in the melting and crystallization temperatures, as well as the degree of crystallinity of PEEK, while simultaneously increasing the glass transition temperature. With increasing comonomer content, a decrease in the elastic modulus and strength in both bending and tension is observed, which is apparently due to amorphization of the material. Upon reaching a DCDPS content of 15%, the mechanical properties reach a plateau and change insignificantly with a further increase in the comonomer concentration. This is because, despite amorphization, the introduction of DCDPS is accompanied by an increase in the macromolecular chain rigidity, as evidenced by an increase in the glass transition temperature. It was established that PEEK and its copolymers are characterized by a similar pattern of change in the G′ and G′ moduli, the values of which increase with increasing frequency. It was shown that the melts of all the studied materials are pseudoplastic liquids, characterized by shear thinning, expressed as a decrease in viscosity with increasing angular velocity. Despite significant differences in viscosity in the low-frequency region, PEEK and its copolymers exhibit similar viscosity values over the shear rate range typical of 3D printing.

It has been shown that copolymers containing DCDPS exhibit improved processability during 3D printing. Unlike the homopolymer, which exhibits warping and delamination from the build plate, the copolymers provide high-quality printing without significant shrinkage. It has been established that the mechanical properties of the printed samples correlate well with their density, which, in turn, is determined by the quality of the filament and the melt viscosity. Overall, the behavior of the mechanical properties of printed copolymer samples with varying DCDPS contents is similar to that of injection-molded samples.

## Figures and Tables

**Table 1:** Monomer ratios during the synthesis of polymers and MFI values.

Sample	DFBN:DCDPS *	MFI, g/10 min
PEEK	1:0	16.9
SPEEK-5	0.95:0.5	21.5
SPEEK-10	0.90:0.10	29.4
SPEEK-15	0.85:0.15	7.5
SPEEK-20	0.80:0.20	8.4
SPEEK-25	0.75:0.25	12.8
SPEEK-50	0.50:0.50	0.3

* DFBN—2.6-difluorobenzonitrile; DCDPS—4,4′-dichlorodiphenylsulfone.

**Table 2:** Thermal properties of PEEK copolymers.

Sample	Tg, °C	T_m_, °C	Tcr, °C	ꭓ, %	T_m_, Onset, °C	T_m_, End, °C	Tcr, Onset, °C	Tcr, End, °C
PEEK	149.9	339.1	290.1	27.9	323.7	345.1	297.4	281.5
5%	153.51	337.5	287.9	18.6	315.8	337.5	294.1	281.5
10%	154.8	325.1	267.1	20.5	311.7	333.1	276.9	258.9
15%	158.1	318.4	264.4	16.3	303.6	325.4	272.1	255.5
20%	161.4	310.1	243.4	15.6	289.5	319.1	268.6	243.4
25%	159.2	300.8	218.1	13.1	281.7	317.6	235.9	196.5
50%	176.9	-	-	-	-	-	-	-

## Data Availability

Data are contained within the article.

## Abbreviations

The following abbreviations are used in this manuscript:

DCDPS	4,4′-dichlorodiphenyl sulfone
DFBP	2,6-difluorobenzophenone
DPS	Diphenylsulfone
DSC	Differential scanning calorimetry
PEEK	Polyetheretherketone
HQ	Hydroquinone
FDM	Fused Deposition Modeling
